# Improvement in quality of life measures in patients with refractory hepatitis C, responding to re-treatment with Pegylated interferon alpha -2b and ribavirin

**DOI:** 10.1186/1477-7525-4-30

**Published:** 2006-05-12

**Authors:** Abraham Mathew, Laurie P Peiffer, Kathy Rhoades, Thomas J McGarrity

**Affiliations:** 1 Division of Gastroenterology and Hepatology, Department of Medicine, Milton S. Hershey Medical Center, Pennsylvania State University, P.O. Box 850, H045 Hershey, 17033-0850 Pennsylvania, USA

## Abstract

**Background:**

In this paper, we report the health related quality of life (HRQOL) data from patients with hepatitis C viral infection (HCV) who were refractory to prior therapy and had re-treatment with a combination of Pegylated interferon alpha-2b and ribavirin. We hypothesized that the HRQOL will improve in those patients who attain sustained viral response similar to naïve patients undergoing treatment for HCV.

**Methods:**

HRQOL data was obtained from 152 patients enrolled into a randomized study for re-treatment of HCV refractory to prior therapy with interferon alpha-2b in combination with ribavirin. The treatment protocol was for 48 weeks and had a high and low dose arm. The HRQOL data was collected at baseline, weeks 24 and 48 of treatment, and at 24 week follow-up after treatment. A repeated measures statistical model was used for comparing the HRQOL domain scores between the responders and non-responders and the treatment groups. The responders and non-responders were also compared to the age and sex adjusted national mean scores.

**Results:**

Twenty-five of the 152 (17%) patients achieved a sustained viral response. At baseline, HRQOL is lower in HCV patients compared to national norms. The norm based HRQOL domain scores for the different domains of the SF-36 instrument were as follows: physical functioning = 47.13, role-physical = 46.87, bodily pain = 48.00, general health = 44.01, vitality = 45.39, social functioning = 47.05, role-emotional = 48.88, mental health = 48.76, physical component score 43.26 and mental component score = 46.17. The scores decreased during therapy in those who would be responders and non-responders, but the pattern of change was different. During the treatment, the HRQOL domain scores of responders decrease notably in the domain of vitality. At week 48 vitality scores were worst in responders. 5 of the 8 domain scores were lower compared to baseline in non-responders. At 24 weeks post treatment follow up, HRQOL in those refractory patients who respond to re-treatment tended to be better than the national average in the domains of vitality (p = .06), social functioning (p = .06) and role-emotional (p = .03) while the non-responders improved their scores in domains of physical function and bodily pain.

**Conclusion:**

We conclude that patients who are to be responders and non-responders behave differently in terms of the HRQOL domain scores when re-treated with a combination of interferon alpha 2b and ribavirin. The responders sustained a significant decrease in the domain score of vitality while 5 of the 8 domain scores decrease in non-responders at the end of treatment. At the end of follow up, in responders, the HRQOL score tended to be better than the national average notably in the domains of role-emotional, vitality and social functioning. On the other hand, in non-responders, the domain scores of physical function improve, while that of role-emotional worsened.

## Background

Hepatitis C virus (HCV) is a common pathogen in the US and globally with a prevalence of 3%. Three million chronically infected persons reside in the US [1-5]. Patients with HCV have a lower Health Related Quality of Life (HRQOL) measure compared to general population, as documented by multiple studies [6-9].

Over the past several years, the treatment of Hepatitis C has evolved [3-5,8,10,11]. The approved therapies at present include monotherapy with different interferon preparations and combination therapy with interferon and ribavirin. The majority of patients are treated for 48 weeks. The treatment for HCV infection inflicts significant side effects on patients and, at best, provides only modest rates (38–43%) of sustained viral suppression [12-16]. The development of pegylated interferon with a slower rate of clearance and a longer half-life has improved the antiviral effects and has decreased the frequency of injections [17-19]. The side effects of interferon-based treatment include fatigue, flu like symptoms, depression, inability to concentrate and decrease in libido. Discontinuation of therapy is commonly due to side effects [7].

A patient who has been previously treated and now has persistent viremia is often considered for re-treatment with a newer interferon or combination treatment. The decision to re-treat a patient for refractory hepatitis C infection is difficult and is influenced by the expected response rates, pattern of response to previous treatment, side-effect profile and effects of treatment on HRQOL. The HRQOL of patients with HCV undergoing naïve treatment is affected significantly by the therapy and has been looked at previously [9,20-22]. In this paper, we report the HRQOL data from patients with HCV infection refractory to prior therapy undergoing re-treatment with a combination of pegylated interferon alpha-2b and ribavirin. We hypothesized that the HRQOL will improve in those patients who attain a sustained viral response, similar to naïve patients undergoing treatment. In this study, 25 of the 152 patients (17%) initiated on the study achieved a sustained viral response. The details of the clinical study and outcomes other than HRQOL measures are reported separately [23,24]

## Methods

HRQOL data was obtained from patients enrolled in a randomized study for treatment of HCV refractory to prior therapy. One hundred and sixty-five patients with refractory Hepatitis C were recruited from the 6 private practice centers and a tertiary care facility, The Milton S. Hershey Medical Center. These patients were enrolled into the open label randomized control study with two arms. In both arms patients received pegylated interferon alpha 2b for 48 weeks (Schering-Plough Institute, Kennelworth, NJ) along with ribavirin, one group at a dose of 1.5 mcg/kg/week (high dose group) and the other at a dose of .5 mcg/kg/week (low dose group). The ribavirin dose was weight based. Patients weighing more that 165 lbs received 1200 mg while those weighing less received 1000 mg.

The entry criteria included chronic hepatitis C documented by a positive HCV RNA, abnormal liver tests and liver biopsy consistent with HCV. Minimum hematological criteria included hemoglobin >10 gm/dl for females and 11 for males, WBC counts >3000/ml and platelet count >70000/ml. Patients with hepatitis B or HIV co-infection, neoplastic disease, severe cardiac or pulmonary disease and psychiatric disorders were excluded. Sustained virologic response was defined as loss of serum HCV RNA at follow-up 24 weeks after discontinuation of treatment.

### Measurement and scoring of quality of life

The HRQOL data was collected using the SF-36 instrument (version 1), which is a widely used and well-validated instrument for measuring HQROL. The data was collected at baseline, 24 weeks, 48 weeks, and at 72 weeks (24 week follow-up after treatment). Each patient score was generated using the tool made available at the official web site for SF-36 . The scores were then transformed into the norm-based scoring and compared to age and sex adjusted national norms. On the norm-based scale, the national mean is 50 and standard deviation is 10 for US general adult population [25].

### Statistical analysis

The changes in HRQOL measures in the different domains of SF-36 instrument during treatment, were assessed at the previously mentioned time points and the data was compared to the baseline. In addition, the responders were compared to the non-responders. In this repeated measures design, a mixed-effects linear model, having an unstructured variance-covariance matrix was fit to each quality of life outcome to assess changes across time (weeks) within and between responder status (responder/non-responder). This was performed using the MIXED procedure of the SAS statistical software package (SAS Institute Inc., Cary, NC). P-values and 95% confidence intervals were adjusted for multiple comparisons using the method of Bonferroni. We also compared the domain scores with the national age and sex adjusted mean domain scores using Student's *t *-test. All dropouts were considered treatment failures.

## Results

One hundred and sixty-five patients were enrolled in the study and randomized into the two arms. After randomization, 13 patients withdrew from the study. Ten of the 13 that withdrew did not complete a baseline questionnaire. Thus at baseline HRQOL data on 155 patients were available. Seventy-eight were in the low dose and 77 were in the high dose arm. Of the 152 patients who proceeded with the study 112 were men (74%) and 40 were female (26%). Twenty-five of the 152 (17%) patients achieved a sustained viral response. The mean age was 46 (range 28 – 69). There were 136 Caucasian (90%), 9 African American, 4 Asian and 3 Hispanic patients. HRQOL data was available from 108 patients during treatment and from 82 patients at the 24 week post treatment follow up. The clinical characteristics of these 152 patients are given in Table 1.

**Table 1:** Clinical characteristics and sustained virologic response rates (SVR)

	**All (SVR ^%^)**	**High dose (SVR^%^)**	**Low dose (SVR^%^)**
**Overall population**	152 (16.5)	72 (21)	80 (12)
**Prior treatment**			
Interferon alone	41 (24)	20 (25)	21 (23)
Interferon and ribavirin	111 (28)	52 (19)	59 (8)
**Hepatitis C Viral genotype**			
1	127 (15)	59 (19)	68 (12)
Non 1	14 (35)	8 (38)	6 (33)
Unavailable	11	5	6
Prior response*			
Non-responder	95 (8)	44 (9)	51 (8)
Relapser	46 (36)	24 (45)	22 (27)
Unavailable	11	4	7

*There was significant difference between the prior non-responders and relapsers in all three groups. p = .0001, .001 & .05 respectively.

At baseline considering all patients, the norm based HRQOL domain scores for the different domains of the SF-36 instrument were as follows: physical functioning = 47.13, role-physical = 46.87, bodily pain = 48.00, general health = 44.01, vitality = 45.39, social functioning = 47.05, role-emotional = 48.88, mental health = 48.76, physical component score 43.26 and mental component score = 46.17. The base lines scores in responders versus non-responders are given in (Table 2). The scores of those who were to be responders were higher, though there was no statistical difference in any of the domains. There was no statistical difference between the two-treatment arms in terms of the HRQOL scores at base line (data not shown).

**Table 2:** Baseline SF-36 norm based Domain scores in refractory HCV patients undergoing re-treatment with peg interferon alpha 2b and ribavirin

**SF-36 domain**	**Responders (**N = 25)	**Non responders **(N = 130)
	Score (SD)	Score (SD)
Physical Function	48.42 (9.8)	46.88 (11.6)
Role Physical	47.46 (13.3)	46.76 (12.6)
Bodily Pain	50.51 (11.3)	47.50 (11.7)
General Health	47.49 (10.4)	43.74 (10.9)
Vitality	48.39 (11.1)	44.80 (11.6)
Social Functioning	49.07 (12.1)	46.66 (11.7)
Role-Emotional	49.49 (11.6)	48.78 (11.4)
Mental Health	50.51 (9.4)	48.42 (9.6)
Physical Component Score	45.47 (9.2)	42.83 (11.3)
Mental Component Score	47.86 (8.2)	45.85 (8.8)

The HRQOL measures were compared to baseline and between the other data points at 24, 48 and 72 weeks (or 24 weeks post treatment) *in responders *and *non-responders *separately (Tables 3 &4). Also, comparisons were made *between *the responders and non-responders and between the treatment groups (Table 5). The results are as follows:

**Table 3:** Change in SF-36 norm based Domain scores in responders among refractory HCV patients re-treated with pegylated interferon alpha 2b and ribavirin

**SF-36 domain**	**Change in QOL scores compared to baseline At**			**48 wks to 24 wks **(24 wk-48 wk)
	24 wks	48 wks	72 wks	
Physical Function	-5.25*	-2.02	1.04	3.23
Role Physical	-7.02	-4.87	5.15	2.14
Bodily Pain	-3.31	-2.13	1.96	1.18
General Health	-4.53*	-3.88	1.55	0.65
Vitality	-8.28**	-8.40^	5.69	-0.12
Social Functioning	-7.33	-5.30	4.87	2.03
Role-Emotional	-4.98	-2.42	5.50	2.56
Mental Health	-4.32	-3.76	0.10	0.56
Physical Component Score	-5.99^^	-3.54	2.26	2.45
Mental Component Score	-5.20*	-3.48	2.49	1.71

*p = .01 to .05, ** p = .001, ^ p = .003, ^^ p = .007. Negative value suggests a decrease in the QOL domain

**Table 4:** Change in SF-36 norm based Domain scores in non-responders among refractory HCV patients re-treated with pegylated interferon alpha 2b and ribavirin

**SF-36 domain**	**Change in scores compared to baseline at**			**48 wks to 24 wks **(24 wk-48 wk)
	24 wks	48 wks	72 wks	
Physical Function	-1.20	-1.37	1.36	0.62
Role-Physical	-4.34	-5.49*	-0.11	-1.16
Bodily Pain	-3.73*	-4.15*	1.32	-0.41
General Health	-1.18	-3.03^	-1.36	-1.84
Vitality	-3.47**	-4.51*	2.05	-1.04
Social Functioning	-3.42	-6.01*	-0.22	-2.59
Role-Emotional	-1.20	-4.42^^	-0.63	-2.42
Mental Health	-1.85	-4.68*	0.58	-2.83
Physical Component Score	-3.34*	-2.83	1.08	0.51
Mental Component Score	-2.13	-3.79*	0.19	-1.65

* p = < .001 to .005, ** p = .006, ^ p = .02, ^^ p = .04. Negative value suggests a decrease in the QOL domain

**Table 5:** Change in SF-36 norm based Domain scores in refractory HCV patients re-treated with pegylated interferon alpha 2b and ribavirin: Responders versus non-responders

**SF-36 domain**	**Change from baseline at **(responders minus non-responders)		
	24 wks	48 wks	72 wks
Physical Function	-3.26	-0.65	-0.32
Role-Physical	-2.68	0.62	5.26
Bodily Pain	0.42	2.02	0.64
General Health	-3.35	-0.85	2.91
Vitality	-4.82	-3.89	3.64
Social Functioning	-3.91	0.71	5.09
Role-Emotional	-2.98	2.00	6.12
Mental Health	-2.47	0.92	-0.48
Physical Component Score	-2.65	-0.71	1.18
Mental Component Score	-3.06	0.30	2.30

None of the changes were statistically significant. Negative value suggest a worse score in responders

### At 24 weeks

At 24 weeks into treatment, in *responders *the HRQOL scores decreased in all domains (Table 3). Statistical significant decrease was noted in the domain of vitality (mean difference -8.23, p = .001). The decrease in general health (mean difference = -4.53 p = .04) and in physical function (mean difference = -5.23, p = .053) were notable but not statistically significant. Among *non-responders*, at 24 weeks, similar to the responders the HRQOL scores decreased. The decrease in HRQOL was significant in the domain of bodily pain (mean difference = -3.7, p = < .001) and borderline significant in the domain of vitality (mean difference = -3.46, p = .006). The decrease in physical role limitation domain was notable, but not significant (mean difference = -4.3, p = .06).

There was no statistically significant difference *between *responders and non-responders comparing the change in the HRQOL scores from baseline (Table 5). The responders had a more prominent decrease in all domains, except bodily pain. There was no statistical difference in the HRQOL scores *between *the high and low dose treatment groups.

### At 48 weeks

At 48 weeks into treatment, in *responders*, the HRQOL score in the domain of vitality remained low similar to week 24, and was significantly lower compared to the baseline (-8.402, p = .003) (Table 3). The scores in the domains of physical function and general health tended to improve from baseline. The *non-responders *on the other hand, sustained further decrease in the domain scores except physical function by 48 weeks compared to the week 24 scores (Table 4). Five of the 8 domain scores were significantly lower from the base line in non-responders at the end of treatment. Physical function, general health and role-emotional were the domains that did not show significant difference compared to the base line

The changes in the HRQOL scores at 48 weeks *between *responders and non-responders were not statistically significant compared to the baseline or week 24 scores (Table 5). No significant changes were noted *between *the high and low dose treatment groups.

### At 72 weeks

At 72 weeks the scores of those who were *responders *improved in all domains, but none were statistically significant compared to the baseline (Table 3). The improvement in vitality among responders, compared to the baseline tended towards statistical significance (5.7, p = .06). Among *non-responders*, the scores at 72 weeks were better than those during the treatment, but were not statistically different from the baseline (Table 4).

At 72 weeks, there was no statistical difference *between *the scores of responders compared to non-responders (Table 5). As before, there were not any changes in the HRQOL scores between the high and low dose treatment arms.

### Comparison to age adjusted national average

Table 6 and 7 show the comparison to age and sex adjusted national mean score for responders and non-responders respectively.

**Table 6:** Norm based SF-36 Domain scores in refractory HCV patients: Comparison to national mean score for responders to re-treatment with peg interferon alpha 2b and ribavirin

**SF-36 domain**	**National age/sex**	**Baseline**		**End of treatment**		**24 wk follow up**	
	adjusted mean	Score	P value	Score	P value	Score	P value
Physical Function	50.18	48.42	.38	46.40	.06	49.47	.72
Role-Physical	50.51	47.46	.13	42.59	.0001	52.61	.29
Bodily Pain	49.69	50.51	.68	48.37	.51	52.47	.16
General Health	50.09	47.49	.20	43.61	.001	49.04	.6
Vitality	50.26	48.39	.35	39.99	.0001	54.08	.06
Social Functioning	50.32	49.07	.58	43.77	.001	53.94	.06
Role-Emotional	50.62	49.49	.60	47.07	.08	54.98	.03
Mental Health	49.57	50.51	.64	46.75	.16	50.61	.60
Physical Component Score	50.16	45.47	.02	41.92	.0001	47.73	.22
Mental Component Score	50.13	47.86	.26	44.37	.004	50.34	.91

**Table 7:** Norm based SF-36 Domain scores in refractory HCV patients: Comparison to national mean score for non-responders to re-treatment with peg interferon alpha 2b and ribavirin

**SF-36 domain**	**National age/sex**	**Baseline**		**End of treatment**		**24 wk follow up**	
	adjusted mean	Score	P value	score	P value	Score	P value
Physical Function	49.62	46.88	<.001	45.51	<.0001	48.25	.10
Role-Physical	50.17	46.76	<.0001	41.26	<.0001	46.64	<.0001
Bodily Pain	49.40	47.51	.03	43.36	<.0001	48.83	.49
General Health	49.82	43.74	<.0001	40.71	<.0001	42.38	<.0001
Vitality	50.29	44.80	<.0001	40.30	<.0001	46.85	<.0001
Social Functioning	50.19	46.66	<.0001	40.64	<.0001	46.44	<.0001
Role-Emotional	50.54	48.78	.04	44.37	<.0001	45.15	.005
Mental Health	49.66	48.42	.14	43.73	.0001	49.00	.67
Physical Component Score	49.64	42.83	<.0001	40.00	<.0001	43.91	<.0001
Mental Component Score	50.32	45.85	<.0001	42.06	<.0001	46.04	.0001

The HRQOL scores of *responders *were not significantly different from the expected age and sex adjusted national mean at baseline. At the completion of treatment week 48 the scores were significantly lower in the domains of role-physical (p = .0001), general health (p = .001), vitality (p = .0001) and social functioning (p = .001). By 24 week post treatment follow up (72 weeks), the scores of responders improved in the domain of role-emotional (p = .03) and tended to improve in the domains of vitality (p = .06) and social functioning (p = .06) compared to the adjusted national mean.

Unlike the responders, the *non-responders *at baseline had HRQOL scores that were significantly lower, compared to an age and sex adjusted national mean in most of the domains. Their scores decreased further through the course of treatment. At 72 weeks the physical function domain score of non-responders, improved such that the score in this domain was no longer significantly different from the national mean score. The domain of bodily pain also showed a tendency to improve. On the other hand the role-emotional domain score decreased further and was significantly lower that the adjusted national mean.

## Discussion

In agreement with previous studies, the HRQOL scores of patients with HCV infection refractory to prior treatment at baseline were low compared to the general population. During the treatment, the scores in many HRQOL domains dropped significantly. In several domains the decline was around 5 points on the norm based scale and hence were likely clinically significant. This is similar to the observation in naïve patients with HCV infection undergoing treatment [20-22,26]. Hassanein et al in their study, showed a decrease in the HRQOL domain scores of those patients treated with Pegylated interferon alfa-2a during the treatment period. They also showed an improvement in several HRQOL domains in those who were responders [22]. They also noted that addition of ribavirin increased the magnitude of decrease in the HRQOL but the pattern of decrease in the HRQOL domains was similar to those patients on interferon only treatment. Similarly, McHutchison et al reported improvement in HRQOL in those patients who achieved sustained virologic response in their study comparing interferon Alfa-2 b monotherapy and combination therapy for naïve HCV patients [21]. In both the above studies [21,22], the domains mostly affected were role-physical, vitality, social functioning and role-emotional. The exact reason for the decline in HRQOL during therapy is unclear. Interferon alone seems to affect the HRQOL domains less [16]. Successful eradication of the virus has been documented previously to cause improvements in the HRQOL, suggesting a causal relationship to HCV infection [9,20].

Curiously, at baseline, the score tended to be better in those who would successfully respond to therapy. Compared to age and sex adjusted national mean scores, the responder group domain scores were not statistically different. On the other hand, the scores of non-responders were significantly lower compared to the national age and sex adjusted average. Why the scores were different and whether it has any bearing on the response to treatment is unclear. It is possible that the prior relapsers have better HRQOL scores (compared to prior non-responders) as the majority of those who responded were prior relapsers. Also, those with better HRQOL may be more likely to tolerate the treatment better and complete the course.

The dose of interferon did not have much bearing on the HRQOL scores. HRQOL scores in patients on the lower dose of interferon were similar to those on the higher dose arm at all the time points.

During the course of treatment, the pattern of change in the HRQOL scores of responders and non-responders was different. At 24 weeks, the decline in vitality scores appeared more dramatic in those who eventually will be responders. More HRQOL domains were affected in those who would be non-responders compared to responders. The scores continued to decrease in non-responders after 24 weeks while in the responders, it stayed steady, if not improved, in all areas except vitality. Vitality measures were significantly affected in both groups and were more pronounced in those who eventually become responders throughout the course of treatment. The scores of the vitality domain were lowest in the responders at the end of treatment. Compared to the baseline the responders sustained a significant decrease in the domain score of vitality, while 5 of the 8 domain scores declined in non responders at the end of treatment.

At the 24 week post-treatment follow up, the change in domain scores from baseline for both responders and non-responders were not statistically significant. However, the scores of responders tended to be better than the national average in the domains of role-emotional, vitality and social functioning. On the other hand the non-responders experienced a decrease in the role-emotional domain scores and had improvement in the physical function and bodily pain domains, such that the scores in these domains were no longer significantly lower from the national mean scores. It is unclear why the score should improve in non-responders. Interestingly, for unclear reasons, the domains with notable change were different in the responder and non-responder groups.

We used a repeated measures design for paired comparisons between the points of observation to do the statistical analysis. The repeated measures model is a powerful statistical model and is especially valuable in a smaller sample. This model nullifies any intra-patient effects on the observations, and hence, the differences noted in the HRQOL are more likely to be real, probably dependent on the effect of treatment. Despite this model, the important limitation of this study is the relatively small sample size and in particular, the low number of responders. The changes of the scores in responders may have been important, but statistical significance might have not been achieved due the small numbers. A sample size of approximately 2000 patients will be required to detect a 2 point change in the norm-based HRQOL scores. It should also be noted that we experienced a significant drop-out rate from the study. Some of the drop-outs were due to side effects, but more often represented patients with persistent viremia at week 24 of therapy who declined further treatment. Chances of clearing the virus if positive at week 24 is slim, and patients were aware of this. All drop-outs were considered as treatment failures in this study. Only 106 (73%) completed 24 weeks of therapy and (88) 58% completed 48 weeks of therapy. We did look at the physical and mental component scores from 24 weeks and did not find a difference between the ones who did and did not drop-out during the course of the study, suggesting that a lower HRQOL was not likely the cause of their dropping out.

## Conclusion

Refractory chronic HCV patients who are to be responders and non-responders behaved differently in terms of the HRQOL domain scores when re-treated with a combination of interferon alpha-2b and ribavirin. Compared to the baseline, the responders sustained a significant decrease in the domain score of vitality while 5 of the 8 domain scores declined in non responders at the end of treatment. Twenty four weeks after the treatment, the HRQOL score in responders tended to be better than the national average notably in the domains of role-emotional, vitality and social functioning. On the other hand, in non-responders, the domain scores of physical function improved while that of role-emotional worsened.

## Competing interests

The authors declare that we have no conflict of interest pertaining to any of the products discussed in this paper. As noted under 'acknowledgments', this paper was partially funded by Scherring Plough Research institute, the manufacturer's of interferon alpha-2b.

## Authors' contributions

AM: Wrote the manuscript, analyzed the HRQOL scores; TJM: Design of the study, collection and co-ordination of data collection; LP: Patient evaluation, Collection and maintenance of data. KR: performed patient evaluations and site visits.
